# Pilot study of eruption forecasting with muography using convolutional neural network

**DOI:** 10.1038/s41598-020-62342-y

**Published:** 2020-03-24

**Authors:** Yukihiro Nomura, Mitsutaka Nemoto, Naoto Hayashi, Shouhei Hanaoka, Masaki Murata, Takeharu Yoshikawa, Yoshitaka Masutani, Eriko Maeda, Osamu Abe, Hiroyuki K. M. Tanaka

**Affiliations:** 1 Department of Computational Diagnostic Radiology and Preventive Medicine, The University of Tokyo Hospital, 7-3-1 Hongo, Bunkyo-ku, Tokyo 113-8655 Japan; 2 Faculty of Biology-Oriented Science and Technology, Kindai University, Nishimitani 930, Kinokawa, Wakayama 649-6493 Japan; 3 Department of Radiology, The University of Tokyo Hospital, 7-3-1 Hongo, Bunkyo-ku, Tokyo 113-8655 Japan; 4 Department of Management, Japan University of Economics, 3-11-25 Gojo, Dazaifu-shi, Fukuoka 818-0197 Japan; 5 Graduate School of Information Sciences, Hiroshima City University, 3-4-1 Ozuka-Higashi, Asaminami-ku, Hiroshima 731-3194 Japan; 6 Earthquake Research Institute, The University of Tokyo, 1-1-1 Yayoi, Bunkyo, Tokyo 113-0032 Japan

**Keywords:** Volcanology, Radiography

## Abstract

Muography is a novel method of visualizing the internal structures of active volcanoes by using high-energy near-horizontally arriving cosmic muons. The purpose of this study is to show the feasibility of muography to forecast the eruption event with the aid of the convolutional neural network (CNN). In this study, seven daily consecutive muographic images were fed into the CNN to compute the probability of eruptions on the eighth day, and our CNN model was trained by hyperparameter tuning with the Bayesian optimization algorithm. By using the data acquired in Sakurajima volcano, Japan, as an example, the forecasting performance achieved a value of 0.726 for the area under the receiver operating characteristic curve, showing the reasonable correlation between the muographic images and eruption events. Our result suggests that muography has the potential for eruption forecasting of volcanoes.

## Introduction

Muography is a newly developed imaging technique utilizing high-energy near-horizontally arriving cosmic muons and enables us to visualize the internal structures of large objects. Muography produces a projection image (hereafter, muogram) of a large body by mapping out the number of muons that are transmitted through it. Muograms were first used in 1970 by Alvarez *et al*. to search for hidden chambers in the Chephren’s Second Pyramid^1^. Almost forty years later, muograms were first used to explore the internal structures of volcanoes^2^. Muograms depicting the internal structures of a volcano are of particular importance because they may be used in the study of eruption dynamics^3^. The first experimental evidence discovered using muograms was obtained by studying the summit of Mount Asama, Japan^2^. The observation, in conjunction with the observation of a low-density magma pathway imaged underneath a solidified magma deposit, confirmed that muograms could resolve a volcano structure with more precision than the preexisting geophysical techniques. Since this first experimental study on the internal structure of volcanos in 2007, similar experiments have been carried not only in Japan^3–6^ but also in the US^7^ and Europe^8–11^.

Eruption forecasting is one of the most critical tasks in modern volcanology^12,13^. For these tasks, many methods based on statistical algorithms or machine learning have been reported^14–19^. These methods use data such as seismic activity, ground deformation, and gas emission. However, to the best of our knowledge, there has been no literature on eruption forecasting using muograms. Muography is conceptually similar to standard X-ray radiography. In the field of medical image analysis, including the analysis of X-ray radiographs, deep learning has been shown to achieve remarkable results^20,21^. We expect that volcanic eruptions can also be forecasted by applying deep learning to muograms.

The purpose of this study is to show the feasibility of eruption forecasting using muograms with deep learning. We focused on muographic data acquired at Sakurajima volcano, Japan, between 2014 and 2016 when it was most activated in the last eruption episode (2009–2017).

## Results

### Muography observation system at Sakurajima volcano

**Figure 1:**
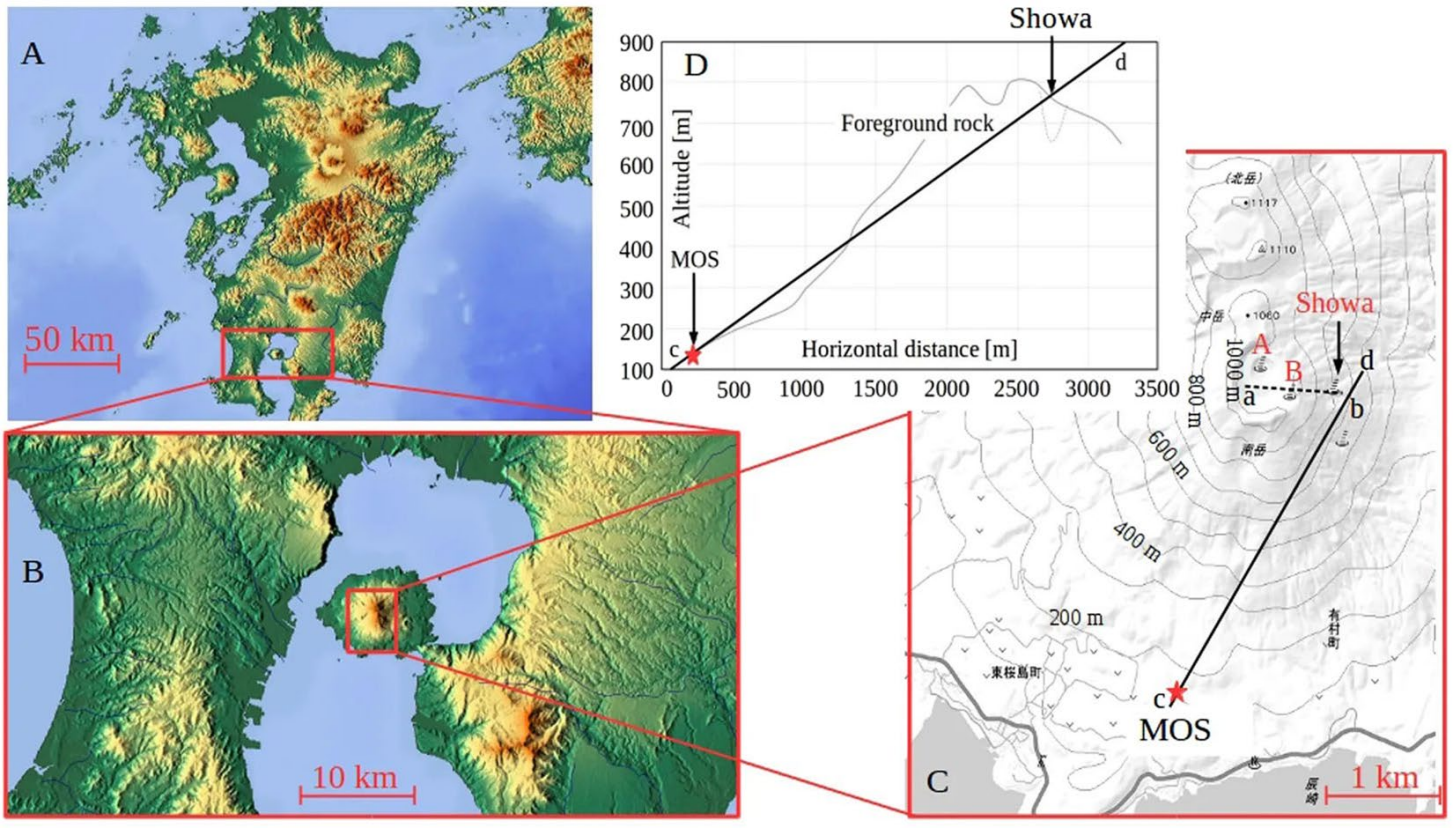
Schematic drawing of our muography experiment. (**A,B**) Location of Sakurajima volcano in Kyushu, Japan (https://maps-for-free.com/). (**C**) Elevation map and the schematic of measurement site. The muography observation system (MOS, red star) was installed in the Southwest direction at distances of 2.8 km, 2.7 km, and 2.6 km from the Showa crater, the Minamidake A crater, and the B crater, respectively. The elevation map was created from data of Geospatial Information Authority of Japan (http://www.gsi.go.jp/) and edited by the authors. (**D**) Cross-sectional view of Sakurajima volcano along line c-d in (**C**). (Source: Oláh, L. *et al*., Sci. Rep. **8**, 3207, pp. 26, cc BY 4.0. We have changed “mMOS” to “MOS” and “Crater Showa” to “Showa”).

The muography observation system (MOS)^4^ was installed at Sakurajima Muography Observatory (SMO)^22^. Figure 1(A–D) respectively show the location of the measurement site, a topographic map of the measurement site, and a cross-sectional view of Sakurajima volcano. A more detailed description of this MOS can be found elsewhere^4^; thus, the MOS will be briefly introduced here. The system consists of five 10-cm-thick lead plates and six layers of scintillation position-sensitive planes. Each position-sensitive plane consists of *N*_*x*_ = 15 and *N*_*y*_ = 15 adjacent scintillator strips, which together form a segmented plane with 15 × 15 segments; thus, the total active area for collecting muons is 2.25 m^2^. The observation system (red star in Fig. 1(C,D)) was installed in the southwest direction at distances of 2.8 km, 2.7 km, and 2.6 km from the Showa crater, the Minamidake A crater, and the B crater, respectively^6^. The three craters were located within the field of view of the MOS. The elevation of the measurement site was ~150 m above sea level (ASL). The angular resolution of the muography observation system was 33 milliradians (mrad).

### Eruption forecasting using convolutional neural network

We investigated the effectiveness of a convolutional neural network (CNN) for eruption forecasting at the Showa crater of Sakurajima volcano based on muograms. The CNN, which is one of the prominent deep learning models, has shown success in image classification and object detection^23^.

**Figure 2:**
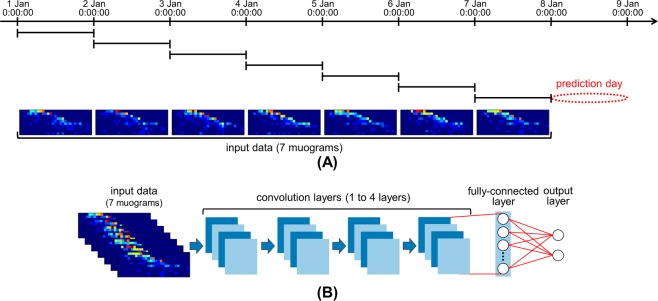
(**A**) Relationship between input data for CNN model and prediction term for eruption, (**B**) configuration of CNN model. Muograms are plotted in a color scale.

Figure 2(A) shows the relationship between the input data for the CNN model and the prediction term for an eruption. The muograms used in this study were plots of the daily muon count (observation period: 00:00:00 to 23:59:59). We inputted muograms obtained for seven consecutive days into the CNN to compute the probability of eruptions for the eighth day, called the “prediction day.” The number of muograms was determined experimentally. If at least one eruption occurred at Showa crater during the prediction day, the day was labeled as an eruption. The eruption times were based on the data from the website of Kagoshima Meteorological Office, Japan (https://www.jma-net.go.jp/kagoshima/vol/kazan_top.html, in Japanese). The total number of eruptions during the prediction day was not referred to. From the above, the CNN model predicts whether an eruption will occur at Showa crater on the eighth day using muograms obtained for seven consecutive days.

We used 464 sets of seven consecutive daily muograms and a label of the prediction day, which were obtained between 7 November 2014 and 12 May 2016. We excluded seven consecutive daily muograms that include unobserved periods due to maintenance of the MOS. We split the dataset into three subsets: a training set, a validation set, and a test set. The training set was used to train the model and included 382 sets of muographic data (prediction days, 20 November 2014–28 January 2016; number of eruption days, 191). The validation set was used to calculate the evaluation criterion of the hyperparameter tuning and includes 40 sets of muographic data (prediction days, 8 February 2016–18 March 2016; number of eruption days, 20). The test set was used to evaluate the best model throughout the hyperparameter tuning, which includes 42 sets of muographic data (prediction days, 25 March 2016–12 May 2016; number of eruption days, 21). During the period of the three datasets, 1,439 and 10 eruptions respectively occurred at the Showa and Minamidake craters.

Figure 2(B) shows the configuration of our network. Our network consisted of one to four convolutional layers, one fully connected layer, and one output layer with two units with softmax activation. We employed a rectified linear unit (ReLU) function^24^ as the activation function for all layers except the output layer. Batch normalization^25^ was performed before each ReLU function. The dropout strategy^26^ was adopted for all layers except the output layer to avoid overfitting. We utilized the Adam method^27^ to optimize the network weights. The training procedure of the CNN model is described in Methods.

We compared the following four input regions:

- Showa crater region: 165 mrad < *θ* <297 mrad, −66 mrad < *ϕ* <66 mrad (5 × 5 segments)
- Minamidake crater region: 198 mrad < *θ* <330 mrad, −330 mrad < *ϕ* <99 mrad (5 × 8 segments)
- surface region: 33 mrad < *θ* <198 mrad, 132 mrad < *ϕ* <264 mrad (5 × 5 segments)
- all segments: 33 mrad < *θ* <330 mrad, −462 mrad < *ϕ* <462 mrad (10 × 29 segments)

where* θ* and *ϕ* indicate the elevation angle and azimuth angle, respectively. The Minamidake crater region included both the Minamidake craters A and B. The surface region included 5 × 5 segments of the mountain surface excluding the three craters as a baseline. The values of the segments outside the volcano were set to 0. For standardization, each segment value was multiplied by 0.001. By assuming the average rock thickness of 1 km in these regions, ~50 and ~90 muon events/day were respectively expected in the Showa and Minamidake crater regions.

**Figure 3:**
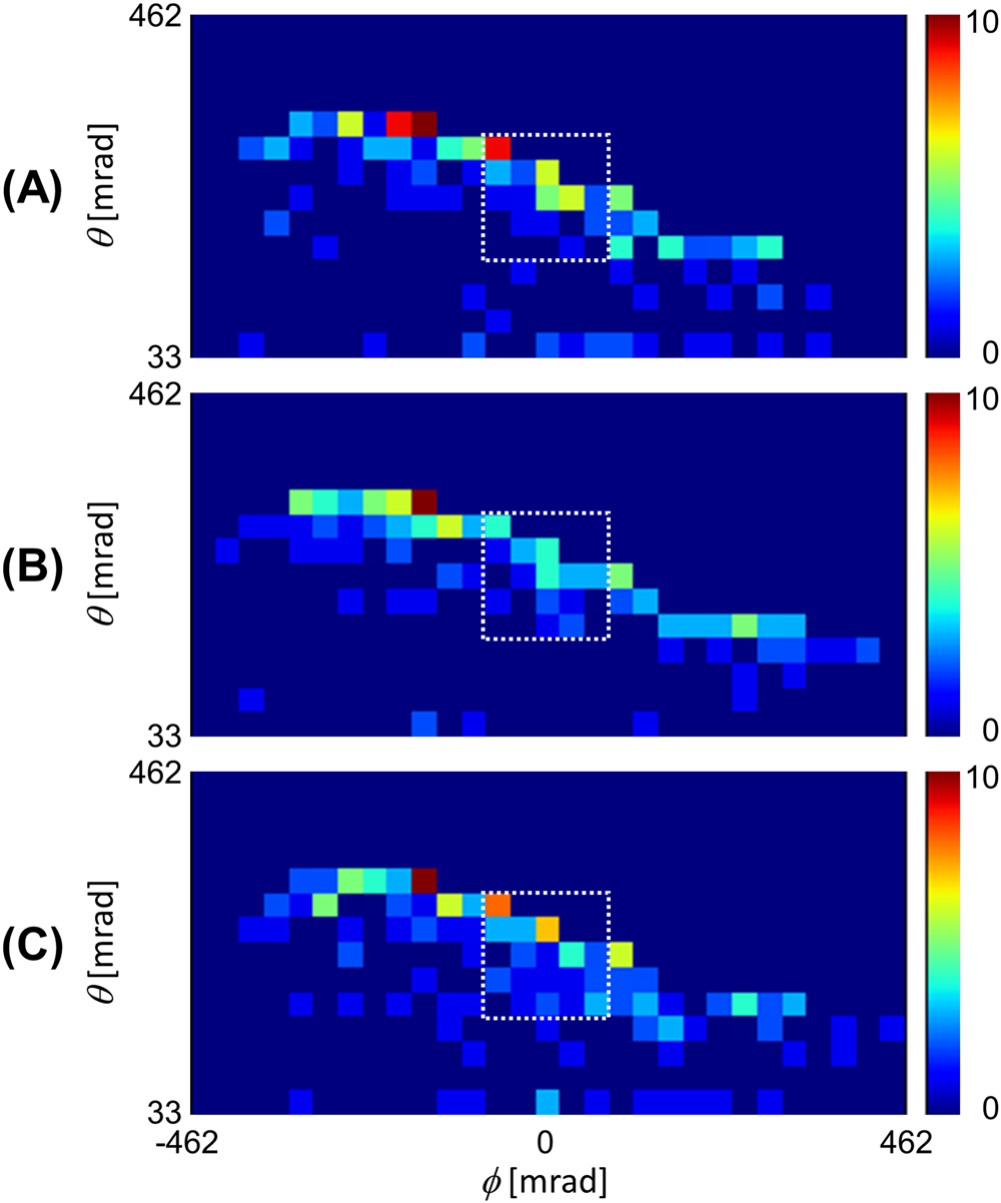
Example of three consecutive daily muograms (observed from (**A**) 25 July 2015 00:00:00 to 23:59:59, (**B**) 26 July 2015 00:00:00 to 23:59:59, (**C**) 27 July 2015 00:00:00 to 23:59:59). The angular resolution was 33 mrad per segment and the data shows 14 × 29 segments. Muograms are plotted in a color scale (range: 0–10). The segment outside of the volcano was set to 0. White dotted squares indicate the Showa crater region. During the observation period, an eruption occurred at 27 June 2015 01:53:00.

**Figure 4:**
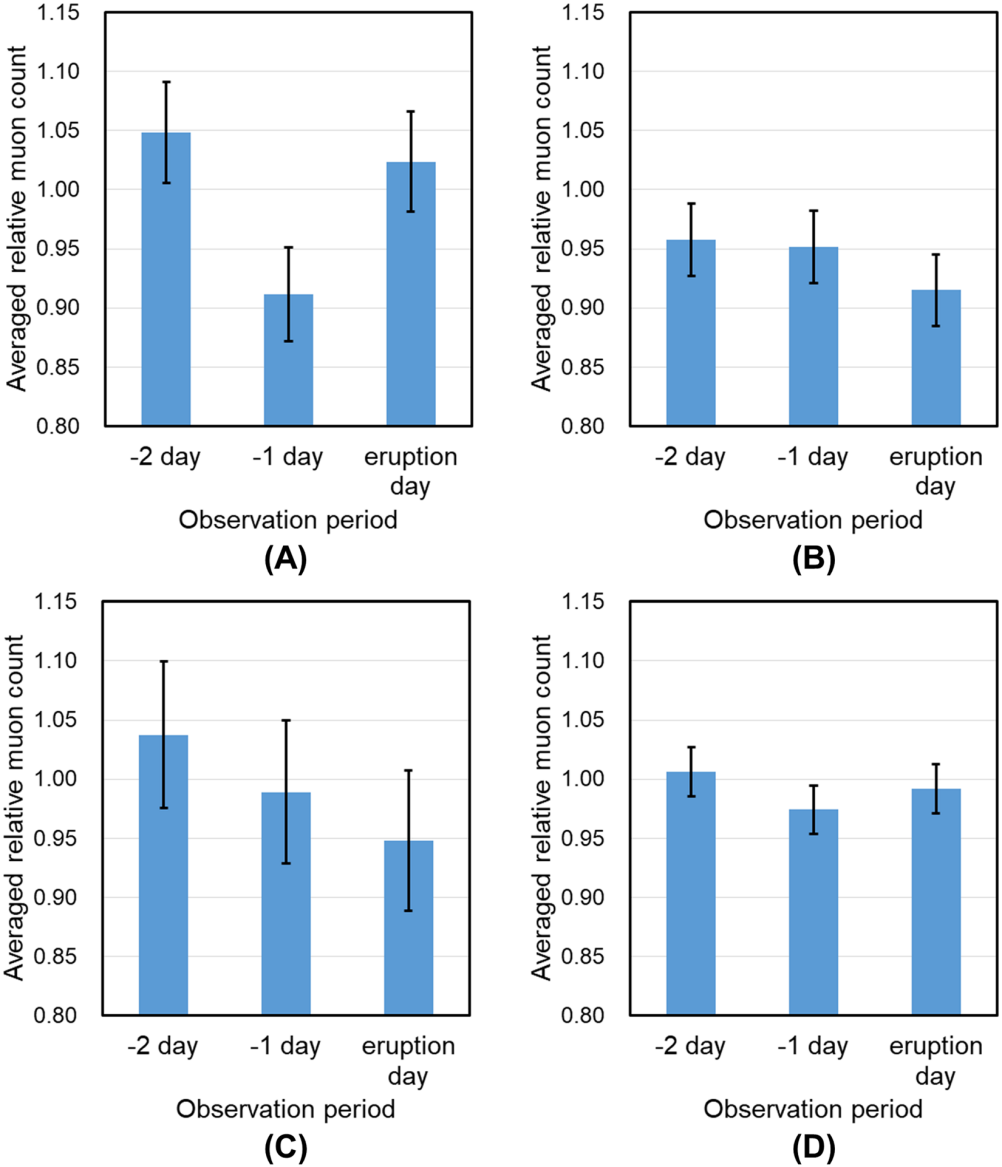
Averaged relative muon counts for each input region ((**A**) Showa crater region, (**B**) Minamidake crater region, (**C**) surface region, (**D**) all segments). Error bars represent standard deviations.

Figure 3 shows an example of three consecutive daily muogram images used for the training set. Figure 4 shows the relative muon counts averaged over 15 events for the training set that were selected to satisfy our condition: there is no eruption at least two days before the eruption, where the “relative muon count” is obtained by dividing by the average of daily muon count acquired during the period of no eruption (from 1 November 2015 to 30 November 2015). The four plots in this figure correspond to the data acquired from the aforementioned four regions ((A) Showa crater region, (B) Minamidake crater region, (C) surface region, and (D) all segments). From Figs. 3,4(A), the muon count of the Showa crater region tended to decrease on the day before the eruption.

For comparison, we also evaluated three types of classifiers: a simple rule-based method, a support vector machine (SVM) model using the radial basis function (RBF) kernel^28^, and a neural network (NN) model. The simple rule-based method classifies an eruption occurring on at least four out of seven consecutive days as an eruption. This threshold was selected to maximize the accuracy of the training data. The SVM model and the NN model were applied only to the Showa crater region. For the feature values of the two models, we compared 175 segment values (25 segment values × 7 days) and seven summed values (the summation of 25 segment values for each day). We selected the seven summed values as the feature values with the best performance. The training procedures of the SVM model and the NN model are described in Methods.

**Figure 5:**
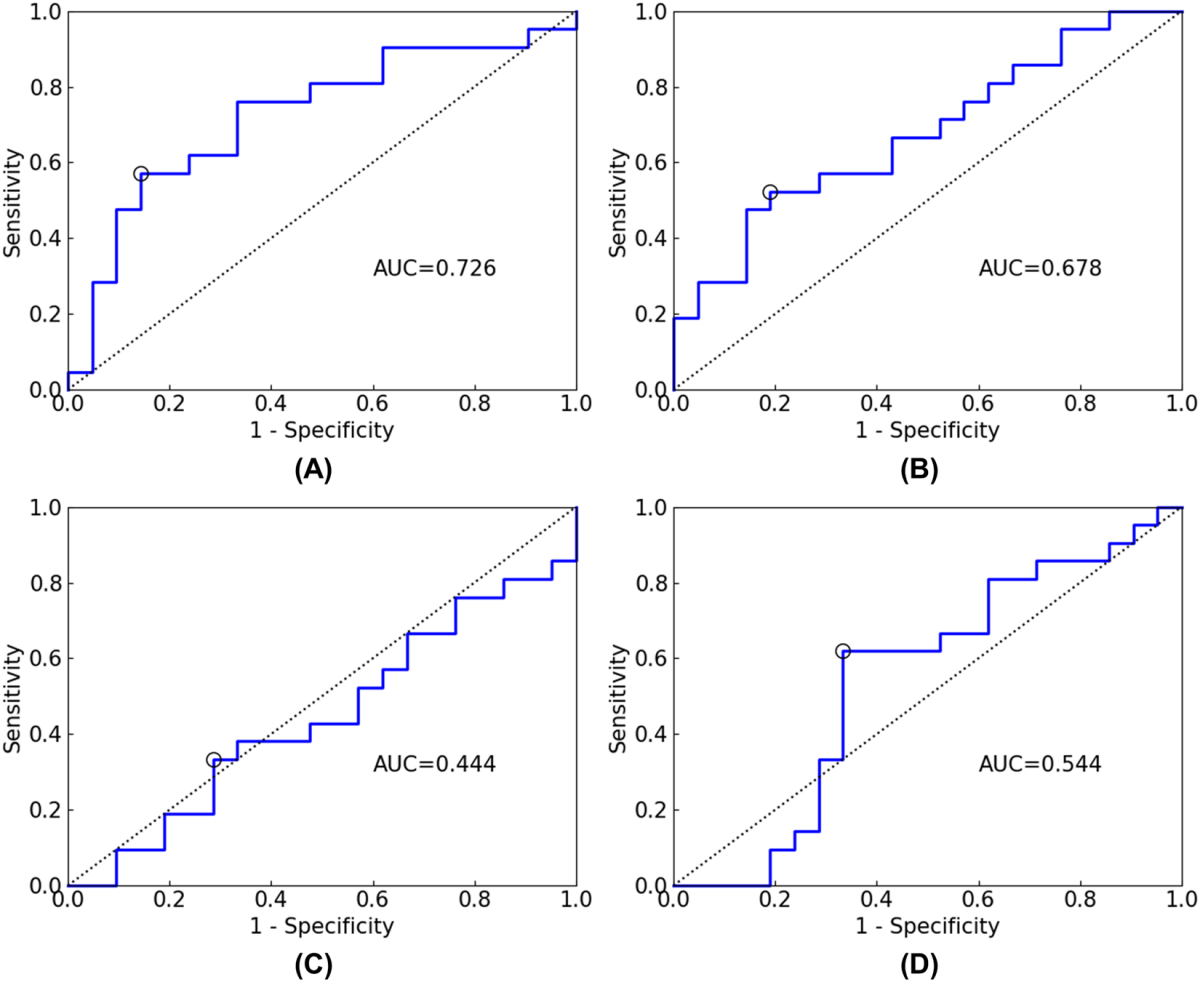
ROC curves for test set for each input data ((**A**) Showa crater region, (**B**) Minamidake crater region, (**C**) surface region, (**D**) all segments). The circle indicates the cutoff point chosen using Youden’s index. AUC is the area under the curve.

**Figure 6:**
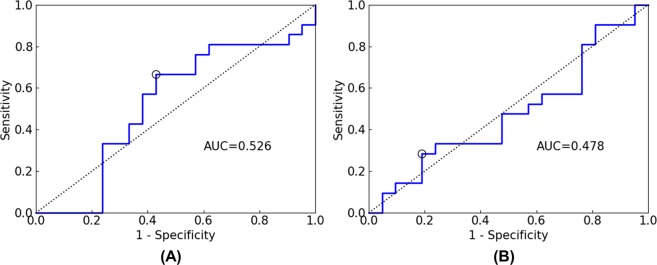
ROC curves for test set for each machine learning model of the Showa crater region ((**A**) SVM, (**B**) NN). The circle indicates the cutoff point chosen using Youden’s index. AUC is the area under the curve.

We evaluated our method using receiver operating characteristic (ROC) analysis^29,30^, and the area under the curve (AUC) was calculated, which ranges between 0.0 and 1.0, with values of 0.5 for random classification and 1.0 for perfect classification. If the AUC is less than 0.5, the classification results are meaningless. Figure 5 shows the ROC curves for the test set for each input data. As shown in Fig. 5, the AUC values were 0.726 for the Showa crater region, 0.678 for the Minamidake crater region, 0.444 for the surface region, and 0.544 for all segments. Figure 6 shows the ROC curves for the test set for the SVM and NN models of the Showa crater. As shown in Fig. 6, the AUC values were 0.569 for the SVM model and 0.499 for the NN model.

Table 1 shows prediction performance at the optimal cutoff point for each input data. The optimal cutoff point of the ROC curve was chosen using Youden’s index^31^. The CNN model of the Showa crater showed the highest accuracy and specificity. In contrast, the SVM model of the Showa crater showed the highest sensitivity.

**Table 1:** Prediction performance at the optimal cutoff point for each input data.

Method	Input region	Accuracy	Sensitivity	Specificity
CNN model	Showa crater	**0.714**	0.571	**0.857**
	Minamidake craters	0.667	0.523	0.810
	Surface	0.524	0.333	0.714
	All segments	0.643	0.619	0.667
Rule-based method		0.571	0.523	0.619
SVM model (Showa crater region)		0.619	**0.667**	0.571
NN model (Showa crater region)		0.548	0.286	0.810

## Discussion

We have shown that our method may achieve moderate performance for day-level eruption forecasting at the Showa crater of Sakurajima volcano. The AUC, accuracy, and specificity were highest when the input to the CNN model was limited to the segments of the Showa crater region. The sensitivity was highest when the input to the CNN model was the all segments. In addition, the muon count of the Showa crater region tends to decrease before the eruption, which was considered to be due to the plugging of a magma pathway by magma deposits on the crater floor. These results suggest that the muographic data of the segments around Showa crater contributed to the forecasting performance. By contrast, the AUC was less than 0.5 when the input to the CNN model was from the segments of the surface region. Consequently, muograms have potential use for eruption forecasting of a volcano by analyzing temporal changes in its internal structures.

The CNN model of the Showa crater region was superior to the SVM model and the NN model in the AUC, accuracy, and specificity. In the CNN model, the convolutional layer works as the feature extractor, extracting local features of each layer. In addition, a deeper convolutional layer can detect more complex features. These roles of the CNN model make it more promising than other machine learning models.

The Showa crater region was limited to 5 × 5 segments since the angular resolution of the muography observation system was 33 mrad per segment. To overcome this problem of low resolution, a high-definition muography observation system was developed, with which muograms have acquired since January 2017^6^. The angular resolution of the high-definition muography observation system is 2.7 mrad per segment, and it can be expected to image more detailed internal structures of the volcano. We plan to investigate the use of high-resolution muograms when a sufficient number of muograms are collected. We also plan to investigate the eruption forecasting of Minamidake craters, because most of the eruptions have occurred from these craters since November 2017.

The consecutive muographic images are time series data. Recurrent neural networks (RNNs), especially long short-term memory (LSTM)^32^, are an effective model for processing time series data^33^. A combination of CNNs and RNNs is expected to improve the forecasting performance. We plan to investigate their combination in our future work.

At Sakurajima volcano, other observation data, such as volcanic earthquake, volcanic tremor, tilt, and GPS data, are reported on the website of Japan Meteorological Agency (http://www.data.jma.go.jp/svd/vois/data/tokyo/STOCK/bulletin/index_vcatalog.html, in Japanese). However, these data are only available until April 2016 at the time of writing. The forecasting performance may be further improved by adding these observations to the input of the CNN.

There are two limitations to this study. First, in the labeling of the eruption day, we have excluded ten eruptions that occurred at the Minamidake craters during the period of analysis. This is because during the training data period, only two eruptions occurred at the Minamidake crater, both of which were erupted at the Showa crater on the same day. Second, an estimation of the uncertainty or confidence in the output decisions of our model was not carried out. For practical eruption forecasting using our method, it is crucial to estimate the uncertainty or confidence of the model^34^.

## Methods

### Training of CNN

In the training of the CNN, the optimization of numerous hyperparameters has a strong effect on the performance of the CNN model. Strategies for hyperparameter optimization include a grid search, random search^35^, and the Bayesian optimization (BO) algorithm^36^. BO is a framework for the optimization of black-box functions whose derivatives and convexity properties are unknown. BO is expected to optimize hyperparameters more efficiently than a random search.

In this study, we carried out 200 trials of hyperparameter tuning with the BO algorithm. The tuned hyperparameters of the CNN were the number of filters of each convolution layer (2^*c*^, *c* = 2–6), the number of units of the fully connected layer (2^*f*^, *f* = 1–7), the batch size, two parameters of the Adam method (*α* = 10^–3^–10^–6^, *β*_1_ = 0.9–0.99), and the ratio of dropout (0–0.5). We utilized the AUC of the ROC curve as an evaluation criterion for hyperparameter tuning. The numbers of maximum epochs and the patience of early stopping^37^ were set to 200 and 20, respectively.

The CNN model was implemented using Keras^38^ version 2.2.4 with TensorFlow^39^ version 1.10.0 backend. We trained the network on a GeForce GTX TITAN X (NVIDIA Corporation, Santa Clara, CA) graphics processing unit (GPU) with 12 GB memory. Table 2 shows the selected sets of hyperparameters of the CNN model for each input data.

**Table 2:** Selected sets of hyperparameters of the CNN model for each input data.

Parameter		Showa crater	Minamidake craters	Surface	All segments
Number of convolution layers		3	2	4	4
Number of filters	first layer	64	8	8	32
	second layer	64	16	8	8
	third layer	4	0	16	64
	fourth layer	0	0	32	8
Number of units of fully connected layer		32	16	64	8
Batch size		8	8	128	16
Parameter of Adam	*α*	0.002455	0.008680	0.002707	0.003291
	*β*_1_	0.991646	0.986270	0.981427	0.998521
Ratio of dropout		0.486933	0.487076	0.325312	0.106837

### Training of SVM

In the training of the SVM model using the RBF kernel, we also carried out 200 trials of hyperparameter tuning with the BO algorithm. The tuned hyperparameters of the SVM model using the RBF kernel were the regularization parameter (*C* = 2^−5^–2^15^) and the kernel parameter (*γ* = 2^−15^–2^3^)^28^. We utilized the AUC of the ROC curve as an evaluation criterion for hyperparameter tuning.

The SVM model was implemented using scikit-learn version 0.21.3. The selected hyperparameters of the SVM model were *C* = 0.0659 and *γ* = 7.237.

### Training of NN

In the training of the NN model, we also carried out 200 trials of hyperparameter tuning with the BO algorithm. The tuned hyperparameters of the NN model were the number of hidden layers (1–3), the number of units of each hidden layer (2^*h*^, *h* = 2–8), the batch size, two parameters of the Adam method (*α* = 10^−3^–10^−6^, *β*_1_ = 0.9–0.99), and the ratio of dropout (0–0.5). We utilized the AUC of the ROC curve as an evaluation criterion for hyperparameter tuning. The maximum number of epochs and the patience of early stopping were set to 100 and 10, respectively.

The NN model was implemented using Keras version 2.2.4 with TensorFlow version 1.10.0 backend. We trained the network on a GeForce GTX TITAN X GPU with 12 GB memory. The selected hyperparameters of the NN model were as follows: number of hidden layers, 3; number of units of first hidden layer, 32; number of units of second hidden layer, 128; number of units of third hidden layer, 16; batch size, 32; *α* of Adam method, 0.000287; *β*_1_ of Adam method, 0.983763; ratio of dropout, 0.442019.

## References

[1] Alvarez LW (1970). Search for hidden chambers in the pyramids. Science.

[2] Tanaka HKM (2007). High resolution imaging in the inhomogeneous crust with cosmic-ray muon radiography: The density structure below the volcanic crater floor of Mt. Asama, Japan. Earth Planet. Sci. Lett..

[3] Tanaka HKM, Kusagaya T, Shinohara H (2014). Radiographic visualization of magma dynamics in an erupting volcano. Nat. Commun..

[4] Tanaka HKM, Uchida T, Tanaka M, Shinohara H, Taira H (2009). Cosmic-ray muon imaging of magma in a conduit: degassing process of Satsuma-Iwojima volcano, Japan. Geophys. Res. Lett..

[5] Tanaka HKM (2016). Instant snapshot of the internal structure of Unzen lava dome, Japan with airborne muography. Sci. Rep..

[6] Oláh L, Tanaka HKM, Ohminato T, Varga D (2018). High-definition and low-noise muography of the Sakurajima volcano with gaseous tracking detectors. Sci. Rep..

[7] Lesparre N (2012). Density muon radiography of La Soufrière of Guadeloupe volcano: comparison with geological, electrical resistivity and gravity data. Geophys. J. Int..

[8] Cârloganu C (2013). Towards a muon radiography of the Puy de Dôme. Geosci. Instrum. Methods Data Syst..

[9] Carbone D (2013). An experiment of muon radiography at Mt Etna (Italy). Geophys. J. Int..

[10] Noli P (2017). Muography of the Puy de Dôme. Ann. Geophys..

[11] Tioukov V (2019). First muography of Stromboli volcano. Sci. Rep..

[12] Amezquita-Sanchez JP, Valtierra-Rodriguez M, Adeli H (2017). Current efforts for prediction and assessment of natural disasters: Earthquakes, tsunamis, volcanic eruptions, hurricanes, tornados, and floods. Sci. Iranica.

[13] Ramis RO (2019). Volcanic and volcano-tectonic activity forecasting: a review on seismic approaches. Ann. Geophys..

[14] Newhall C, Hoblitt R (2002). Constructing event trees for volcanic crises. Bull. Volcanol..

[15] Marzocchi W, Sandri L, Selva J (2008). BET_EF: a probabilistic tool for long- and short-term eruption forecasting. Bull. Volcanol..

[16] Lindsay J (2010). Towards real-time eruption forecasting in the Auckland Volcanic Field: application of BET_EF during the New Zealand National Disaster Exercise ‘Ruaumoko’. Bull. Volcanol..

[17] Segall P (2013). Volcano deformation and eruption forecasting. Geol. Soc., London, Spec. Publ..

[18] Brancato, A., Buscema, P., Massini, G. & Gresta, S. Pattern recognition for flank eruption forecasting: an application at Mount Etna volcano (Sicily, Italy). *Open J. of Geol*. (2016).

[19] Brancato A (2019). K-CM application for supervised pattern recognition at Mt. Etna: an innovative tool to forecast flank eruptive activity. Bull. Volcanol..

[20] Litjens G (2017). A survey on deep learning in medical image analysis. Med. Image Anal..

[21] Sahiner B (2019). Deep learning in medical imaging and radiation therapy. Med. Phys..

[22] Oláh L, Tanaka HKM, Ohminato T, Hamar G, Varga D (2019). Plug formation imaged beneath the active craters of Sakurajima volcano with muography. Geophys. Res. Lett..

[23] LeCun Y, Bengio Y, Hinton G (2015). Deep learning. Nature.

[24] Nair, V. & Hinton, G. E. Rectified linear units improve restricted Boltzmann machines. *Proceedings of the 27th International Conference on Machine Learning*. 807–814 (2010).

[25] Ioffe, S. & Szegedy, C. Batch normalization: accelerating deep network training by reducing internal covariate shift. *arXiv preprint*. arXiv:1502.03167 (2015).

[26] Srivastava N, Hinton G, Krizhevsky A, Sutskever I, Salakhutdinov R (2014). Dropout: a simple way to prevent neural networks from overfitting. J. Mach. Learn. Res..

[27] Kingma, D. P. & Ba, J. Adam: a method for stochastic optimization. *arXiv preprint*. arXiv:1412.6980 (2014).

[28] Hsu, C. W., Chang, C. C. & Lin, C. J. A practical guide to support vector classification, http://www.csie.ntu.edu.tw/~cjlin/papers/guide/guide.pdf (2016).

[29] Obuchowski NA (2005). ROC analysis. AJR Am. J. Roentgenol..

[30] Fawcett T (2006). An introduction to ROC. analysis. Pattern Recognit. Lett..

[31] Youden WJ (1950). Index for rating diagnostic tests. Cancer.

[32] Hochreiter S, Schmidhuber J (1997). Long short-term memory. Neural Comput.

[33] Chen J, Yang L, Zhang Y, Alber M, Chen DZ (2016). Combining fully convolutional and recurrent neural networks for 3D biomedical image segmentation. In Advances in Neural Information Processing Systems.

[34] Kendall A, Gal Y (2017). What uncertainties do we need in bayesian deep learning for computer vision?. In Adv. Neural Inf. Process. Syst..

[35] Bergstra J, Bengio Y (2012). Random search for hyper-parameter optimization. J. Mach. Learn. Res..

[36] Snoek J, Larochelle H, Adams RP (2012). Practical Bayesian optimization of machine learning algorithms. In Adv. Neural Inf. Process. Syst..

[37] Prechelt, L. Early stopping – but when? In *Neural Networks: Tricks of the Trade: Second Edition* (eds. Montavon, G., Orr, G. B. & Müller, K.-R.) 53–67 (Springer, Berlin, Heidelberg, 2012).

[38] Chollet, F. *et al*. Keras, https://www.keras.io (2015).

[39] Abadi, M. *et al*. TensorFlow: Large-scale machine learning on heterogeneous distributed systems. *arXiv preprint*. arXiv:1603.04467 (2016).

